# Hospital Discharge Rates Before and After Implementation of a City-wide Smoking Ban in a Texas City, 2004–2008

**DOI:** 10.5888/pcd9.120079

**Published:** 2012-12-27

**Authors:** Phil Head, Bradford E. Jackson, Sejong Bae, Debra Cherry

**Affiliations:** Author Affiliations: Phil Head, Occupational Medical Care, Houston, Texas; Bradford E. Jackson, Sejong Bae, University of Alabama at Birmingham, Birmingham, Alabama. At the time of the study, Dr Head was at the University of Texas Health Science Center at Tyler, Tyler, Texas.

## Abstract

The objective of this study was to examine hospital discharge data on 5 tobacco-related diagnoses before and after implementation of a smoking ban in a small Texas city. We compared hospital discharge rates for 2 years before and 2 years after implementation of the ban in the intervention city with discharge rates during the same time in a similar city with no ban. The discharge rates for blacks and whites combined declined significantly after the ban in the intervention city for acute myocardial infarction (MI) (rate ratio [RR], 0.74; 95% confidence interval [CI], 0.65–0.85) and for stroke or cerebrovascular accident (RR, 0.71; 95% CI, 0.62–0.82); discharge rates in the intervention city also declined significantly for chronic obstructive pulmonary disease (RR, 0.64; 95% CI, 0.54–0.75) and asthma (RR, 0.69; 95% CI, 0.52–0.91) for whites only. Discharge rates for 4 of 5 diagnoses in the control city did not change. Although postban reduction in acute MI is well documented, this is one of the first studies to show a racial disparity in health benefits and a decline in tobacco-related diagnoses other than acute MI after implementation of a city-wide smoking ban.

## Objective

The 2006 Surgeon General’s report on the health effects of secondhand smoke on nonsmokers implicated even limited smoke exposure as an etiological factor in heart diseases, lung diseases, cerebral vascular events, and sudden infant death (1). Smoking bans lead to dramatic and immediate reductions in acute myocardial infarction (MI) (2), but the effect on other diagnoses is not well documented. The objective of this study was to examine hospital discharge data on 5 tobacco-related diagnoses before and after implementation of a smoking ban in Beaumont, Texas.

## Methods

We compared hospital discharge rates from July 2004 through June 2006 (before the ban) with hospital discharge rates from July 2006 through June 2008 (after the ban) in Beaumont, Texas, which implemented a smoking ban in July 2006. We compared the rates in Beaumont with rates in Tyler, Texas, which had no smoking restrictions during this period. We compared rates in Beaumont and Tyler with rates for all Texas hospitals, which reflected a mix of smoking policies. We examined discharge rates for 5 tobacco-related diagnoses and for all diagnoses (including nontobacco-related diagnoses). The Beaumont ban prohibited smoking in all public places, including municipal workplaces, private-sector workplaces, and all restaurants and bars. Tyler and Beaumont are in East Texas and have similar demographic characteristics, although Beaumont has a larger non-Hispanic black population (Table 1).

**Table 1:** Demographic Characteristics of Tyler, Texas, and Beaumont, Texas, 2005 and 2008

Characteristic	Tyler (Control)		Beaumont (Ban)	
	2005	2008	2005	2008
Total population,^a^n	87,687	87,099	107,876	115,053
Non-Hispanic white only, n (%)	43,427 (50)	48,444 (56)	36,288 (34)	42,180 (37)
Non-Hispanic black only, n (%)	24,837 (28)	21,114 (24)	53,829 (50)	58,217 (51)
Hispanic, n (%)	17,076 (19)	15,955 (18)	12,088 (11)	10,257 (9)
Median age, y	33.8	34.7	33.1	33.5
Median annual household income, $	45,644	43,545	43,014	48,747

a Sources: US Census Bureau (3).

The Texas Department of State Health Services provided discharge data from the Texas Hospital Inpatient Discharge Public Use Data File by quarter from July 2004 through June 2008 (4). The numerator for discharge rates in Tyler and Beaumont was defined as 1) one of 3 hospitals in Tyler or one of 2 in Beaumont; 2); for each person representing a discharge, a residential zip code within Tyler or Beaumont; 3) a hospital discharge date from July 2004 through June 2008; and 4) a discharge diagnosis based on the *International Classification of Diseases, 9th Revision, Clinical Modification* (ICD-9-CM) of 410.xx (acute MI); 434.xx (stroke or cerebrovascular accident [CVA]); 435.xx (transient ischemic attack [TIA]); 491.xx, 492.xx, or 496.xx (chronic obstructive pulmonary disease [COPD]); or 493.xx (asthma). The denominator was the intercensal estimate of the city population for each year (3). We calculated population rates per 100,000. For the Texas rates, the numerator was defined by the same ICD-9-CM codes, and the denominator was the intercensal estimate of the state population for each year. The numerator for Texas rates was not limited to Texas residents.

We also examined discharge rates by non-Hispanic black and non-Hispanic white race. We calculated rate ratios for hospital discharges before and after the ban for each diagnosis in Beaumont, Tyler, and all Texas, using the before-ban rate as the denominator and the after-ban rate as the numerator. A rate ratio less than 1.0 represents a decrease in rate. We calculated rate ratios using SAS version 9.3 (SAS Institute, Inc, Cary, North Carolina). The study was approved as exempt by the institutional review board at the University of Texas Health Science Center at Tyler.

## Results

Hospital discharges declined significantly for blacks, whites, and both races combined for acute MI, CVA, and total discharges for all diagnoses in Beaumont after the ban (Table 2 and Table 3). For all races combined, acute MI discharges declined by 26% and CVA declined by 29%. For whites only, COPD discharges declined by 36%, and asthma declined by 31%. The discharge rates for 4 of 5 diagnoses in the control city did not change. The total discharge rate declined by 4% in Beaumont, by 6% in Tyler, and by 6% in Texas. The baseline discharge rate (2004–2006) for all diagnoses combined was higher in Beaumont (35,943 per 100,000) than in Tyler (26,182 per 100,000). 

**Table 2:** Discharge Rates **a** for Selected Diagnoses Before and After Implementation of a Smoking Ban in Beaumont, Texas, Compared With Rates in Tyler, Texas (No Ban), and All Texas (Mixed Policies)

Diagnosis (ICD-9-CM code)	Rate (No.)					
	Before Ban (July 2004–June 2006)			After Ban (July 2006–June 2008)		
	All	Non-Hispanic Black	Non-Hispanic White	All	Non-Hispanic Black	Non-Hispanic White
**Beaumont, Texas (Ban)**						
Acute myocardial infarction (410.xx)	461.3 (514)	365.9 (197)	782.6 (284)	342.4 (376)	250.8 (146)	490.8 (207)
Stroke or cerebrovascular accident (434.xx)	470.3 (524)	458.9 (247)	697.2 (253)	336.0 (369)	345.3 (201)	367.5 (155)
Transient ischemic attack (435.xx)	266.5 (297)	245.2 (132)	410.6 (149)	246.8 (271)	213.0 (124)	329.5 (139)
Chronic obstructive pulmonary disease (491.xx, 492.xx, or 496.xx)	482.8 (538)	356.7 (192)	923.2 (335)	426.2 (468)	371.0 (216)	587.9 (248)
Asthma (493.xx)	343.7 (383)	425.4 (229)	330.7 (120)	339.7 (373)	427.7 (249)	227.6 (96)
Total hospital discharges for all diagnoses^b^	35,943.6 (40,052)	32,798.3 (17,655)	52,857.7 (19,181)	34,420.0 (37,797)	28,266.7 (16,456)	42,766.7 (18,039)
City population	111,430	53,829	36,288	109,808	58,217	42,180
**Tyler, Texas (No Ban)**						
Acute myocardial infarction (410.xx)	346.7 (313)	249.6 (62)	550.4 (239)	359.0 (346)	322.1 (68)	534.6 (259)
Stroke or cerebrovascular accident (434.xx)	392.1 (354)	434.8 (108)	518.1 (225)	286.4 (276)	421.5 (89)	365.4 (177)
Transient ischemic attack (435.xx)	82.0 (74)	64.4 (16)	128.9 (56)	78.9 (76)	85.3 (18)	115.6 (56)
Chronic obstructive pulmonary disease (491.xx, 492.xx, or 496.xx)	254.8 (230)	136.9 (34)	449.0 (195)	222.1 (214)	156.3 (33)	367.4 (178)
Asthma (493.xx)	197.2 (178)	346.3 (86)	179.6 (78)	189.9 (183)	421.5 (89)	161.0 (78)
Total hospital discharges for all diagnoses^b^	26,182.5 (23,636)	25,981.4 (6,453)	31,913.3 (13,859)	24,574.6 (23,683)	29,781.2 (6,288)	28,137.6 (13,631)
City population	90,274	24,837	43,427	96,372	21,114	48,444
**All Texas (Mixed Policies)**						
Acute myocardial infarction (410.xx)	360.7 (80,322)	336.4 (8,102)	463.9 (50,554)	323.7 (78,740)	302.8 (8,273)	433.0 (49,745)
Stroke or cerebrovascular accident (434.xx)	232.4 (51,747)	365.6 (8,806)	273.5 (29,810)	210.1 (51,104)	312.5 (8,538)	258.2 (29,656)
Transient ischemic attack (435.xx)	99.0 (22,025)	121.5 (2,926)	125.4 (13,669)	91.0 (22,134)	105.5 (2,884)	122.9 (14,122)
Chronic obstructive pulmonary disease (491.xx, 492.xx, or 496.xx)	302.9 (67,465)	256.6 (6,180)	468.1 (51,018)	292.9 (71,264)	245.8 (6,716)	473.9 (54,444)
Asthma (493.xx)	235.7 (52,493)	508.1 (12,239)	209.8 (22,860)	229.4 (55,794)	473.4 (12,936)	208.9 (24,004)
Total hospital discharges for all diagnoses^b^	25,636.3 (5,709,241)	29,679.4 (714,885)	26,925.7 (2,934,527)	24,073.8 (5,856,424)	27,303.8 (746,093)	26,334.4 (3,025,311)
State population	22,270,165	2,408,694	10,898,613	24,326,974	2,732,563	11,488,049

Abbreviation: ICD-9-CM, *International Classification of Disease, Ninth Revision, Clinical Modification*.

a Discharge rate per 100,000 population calculated as the number of discharges per diagnosis matched to patient who reported a residential zip code in either of 2 cities (Beaumont or Tyler) and was admitted to a hospital in his or her own city during the 2-year study period divided by the average intercensal estimate of the city population for the same period multiplied by 100,000.

b Includes nontobacco-related discharges.

**Table 3:** Discharge Rate Ratios^a^ and Confidence Intervals for Selected Diagnoses Before and After Implementation of a Smoking Ban in Beaumont, Texas, Compared With Rate Ratios in Tyler, Texas (No Ban), and All Texas (Mixed Policies)

Diagnosis (ICD-9-CM code)	Rate Ratio (95% CI)		
	All	Non-Hispanic Black	Non-Hispanic White
**Beaumont, Texas (Ban)**			
Acute myocardial infarction (410.xx)	0.74 (0.65–0.85)^b^	0.68 (0.55–0.85)^b^	0.63 (0.52–0.75)^b^
Stroke or cerebrovascular accident (434.xx)	0.71 (0.62–0.82)^b^	0.75 (0.62–0.91)^b^	0.53 (0.43–0.65)^b^
Transient ischemic attack (435.xx)	0.92 (0.78–1.09)	0.87 (0.67–1.12)	0.80 (0.63–1.02)
Chronic obstructive pulmonary disease (491.xx, 492.xx, or 496.xx)	0.88 (0.78–1.00)	1.04 (0.85–1.27)	0.64 (0.54–0.75)^b^
Asthma (493.xx)	0.98 (0.85–1.14)	1.00 (0.84–1.21)	0.69 (0.52–0.91)^b^
Total hospital discharges for all diagnoses^c^	0.96 (0.94–0.97)	0.86 (0.84–0.88)^b^	0.81 (0.79–0.83)^b^
**Tyler, Texas (No Ban)**			
Acute myocardial infarction (410.xx)	1.04 (0.89–1.21)	1.29 (0.90–1.85)	0.97 (0.81–1.16)
Stroke or cerebrovascular accident (434.xx)	0.73 (0.62–0.86)^b^	0.97 (0.72–1.29)	0.71 (0.58–0.86)^b^
Transient ischemic attack (435.xx)	0.96 (0.69–1.34)	1.32 (0.64–2.77)	0.89 (0.61–1.32)
Chronic obstructive pulmonary disease (491.xx, 492.xx, or 496.xx)	0.87 (0.72–1.05)	1.14 (0.69–1.89)	0.82 (0.66–1.01)
Asthma (493.xx)	0.96 (0.78–1.19)	1.22 (0.89–1.66)	0.89 (0.65–1.24)
Total hospital discharges for all diagnoses^c^	0.94 (0.92–0.96)	1.15 (1.11–1.19)	0.88 (0.86–0.90)^b^
**All Texas (Mixed Policies)**			
Acute myocardial infarction (410.xx)	0.89 (0.89–0.91)^b^	0.90 (0.87–0.93)	0.93 (0.92–0.95)
Stroke or cerebrovascular accident (434.xx)	0.90 (0.89–0.92)	0.85 (0.83–0.88)^b^	0.94 (0.93–0.96)
Transient ischemic attack (435.xx)	0.92 (0.90–0.94)	0.87 (0.83–0.92)^b^	0.98 (0.96–1.00)
Chronic obstructive pulmonary disease (491.xx, 492.xx, or 496.xx)	0.97 (0.96– 0.98)	0.96 (0.93–0.99)	1.01 (1.00–1.02)
Asthma (493.xx)	0.97 (0.96–0.98)	0.93 (0.91–0.96)	0.99 (0.98–1.01)
Total hospital discharges for all diagnoses^c^	0.94 (0.93–0.94)	0.92 (0.92–0.92)	0.98 (0.97–0.98)

Abbreviations: ICD-9-CM, *International Classification of Disease, Ninth Revision, Clinical Modification*; CI, confidence interval.

a Ratio calculated by using the before-ban rate as the denominator and the after-ban rate as the numerator. A rate ratio less than 1.0 represents a decrease in discharge rate. The lower the rate ratio, the more the after-ban discharge rate declined relative to the before-ban discharge rate.

b *P* < .05 and rate ratio < 0.9.

c Includes nontobacco-related discharges.

## Discussion

Although we attribute the decline in tobacco-related discharge rates in Beaumont to the smoking ban, we considered alternative explanations. The discharge rates excluded people who resided outside Beaumont but may have worked in Beaumont. Excluding these people would be expected to weaken the study’s power to detect health benefits from the ban, but discharge rates declined despite this. We considered that hospital business or the population overall may have declined after hurricanes Katrina and Rita in 2005; however, the population of Beaumont barely changed after 2006, and the discharge rate for all diagnoses declined by only 4%, compared to a 6% decline for Texas during the same time. Perhaps a change in referral patterns, such as acute MIs being treated outside Beaumont after the ban, lowered tobacco-related discharge rates; however, both Beaumont hospitals continued to treat acute MI, stroke, and COPD after the ban. The slight decline in total discharges for all diagnoses in Beaumont cannot explain the larger decline in discharges for tobacco-related diagnoses. Differences in insurance coverage, outpatient and inpatient procedures, and admission criteria could explain the slight decline in total rates.

We were surprised that asthma and COPD discharges declined only among whites in Beaumont. Perhaps the ban had a greater effect on whites because of their heavier smoking habits, if East Texas has the same racial pattern of smoking habits as other parts of the country. No data from the Behavioral Risk Factor Surveillance System (BRFSS) or the National Health Interview Survey on tobacco use are available at the city or county levels for Beaumont (Jefferson County) or Tyler (Smith County); however, modeled data indicate that about 22% of adults were current smokers in Jefferson and Smith counties in 2011 (5). Texas BRFSS data indicate a higher prevalence of current smokers among blacks (23%) than whites (20%) during 2005 through 2008 (6) but provide no information about the number of cigarettes smoked by race. Nationally, however, in 2005 through 2007, white smokers averaged 5 more cigarettes per day than black smokers, and white male smokers were nearly 4 times as likely as black male smokers to smoke 35 cigarettes or more per day (7).

This study had several limitations. The ecological analysis cannot establish a causal relationship between the ban and discharges. Follow-up studies evaluating individual risk factors (eg, case-control), including active and passive smoke exposure of discharged patients, would help explain the association between the ban and declines in discharges.

Although post-ban reduction in acute MI is well documented (2), this is one of the first studies to show a racial disparity in health benefits and a decline in tobacco-related diagnoses other than acute MI after implementation of a city-wide smoking ban.

## References

[1] US Department of Health and Human Services. The health consequences of involuntary exposure to tobacco smoke: a report of the Surgeon General. http://www.surgeongeneral.gov/library/secondhandsmoke/. Accessed February 24, 2012. 20669524

[2] Meyers DG , Neuberger JS , He J . Cardiovascular effect of bans on smoking in public places: a systematic review and meta-analysis. J Am Coll Cardiol 2009;54(14):1249–55. 10.1016/j.jacc.2009.07.022 19778665

[3] US Census Bureau. American Community Survey. 1-year estimate data sets for 2005 and 2008. http://factfinder2.census.gov/faces/nav/jsf/pages/index.xhtml#none, expand “topics” and enter year = 2005 or 2008, geography = Beaumont City TX or Tyler TX; retrieve data from multiple tables; example for Beaumont 2005: http://factfinder2.census.gov/faces/tableservices/jsf/pages/productview.xhtml?pid=ACS_05_EST_DP1&prodType=table. Accessed October 1, 2012.

[4] Department of State Health Services, Center for Health Statistics. Texas hospital inpatient discharge public use data file. http://www.dshs.state.tx.us/thcic/hospitals/Inpatientpudf.shtm. Accessed September 19, 2012.

[5] University of Wisconsin Population Health Institute. County health rankings and roadmaps. http://www.countyhealthrankings.org/. Accessed September 19, 2012.

[6] Texas Department of State Health Services. Behavioral Risk Factor Surveillance System data table lookup. http://www.dshs.state.tx.us/chs/brfss/query/brfss_form.shtm. Accessed September 19, 2012.

[7] Schoenborn CA , Adams PF . Health behaviors of adults: United States, 2005–2007. Vital Health Stat 10 (245); Hyattsville (MD): National Center for Health Statistics; 2010. http://www.cdc.gov/nchs/data/series/sr_10/sr10_245.pdf. Accessed July 28, 2012. 20669609

